# Migrant female head porters’ enrolment in and utilisation and renewal of the National Health Insurance Scheme in Kumasi, Ghana

**DOI:** 10.1007/s10389-017-0832-1

**Published:** 2017-09-13

**Authors:** Simon Boateng, Prince Amoako, Adjoa Afriyie Poku, Anthony Baabereyir, Razak Mohammed Gyasi

**Affiliations:** 1 Social Sciences Department, St. Monica’s College of Education, Mampong, Ghana; 2 Department of Economics, Kwame Nkrumah University of Science and Technology, Kumasi, Ghana; 3 Department of Geography Education, University of Education, Winneba, Ghana; 4 Department of Sociology and Social Policy, Faculty of Social Sciences, Lingnan University, Teun Mun, Hong Kong

**Keywords:** National Health Insurance Scheme, Migrant female head porters, Enrolment, Ethnic minorities, Utilisation, Renewal

## Abstract

**Purpose:**

As a social protection policy, Ghana’s National Health Insurance Scheme (NHIS) aims to improve access to healthcare, especially for the vulnerable. Migrant female head porters (*kayayoo*), who are part of the informal economic workforce, are underscored as an ethnic minority and vulnerable group in Ghana. This study aimed to analyse the factors associated with enrolment in and renewal and utilisation of the NHIS among migrant female head porters in the Kumasi Metropolis.

**Method:**

We purposively sampled 392 migrant female head porters in the Kejetia, Asafo and Bantama markets. We used a binary logit regression model to estimate associations among baseline characteristics, convenience and benefit factors and enrolment in and renewal and utilisation of the NHIS.

**Result:**

Age and income significantly increased the probability of NHIS enrolment, renewal and utilisation. Long waiting times at NHIS offices significantly reduced the likelihood of renewal, while provision of drugs highly significantly increased the tendency for migrant female head porters to enrol in, renew and use the NHIS. Consulting and surgery also significantly increased renewal and utilisation of the NHIS.

**Conclusion:**

Political commitment is imperative for effective implementation of the decentralisation policy of the NHIS through the National Health Insurance Authority in Kumasi. We argue that retail offices should be well equipped with logistic facilities to ensure convenience in NHIS initial enrolment and renewal processes by citizenry, and by vulnerable groups in particular.

## Introduction

Human migration and the associated public health challenges are not new global phenomena. According to the 2015 World Migration Report, about 224 million people worldwide are on the move, 49% of whom are women (UNDESA 2016). Stressful economic conditions, poverty and unemployment, protracted ethnic conflicts, income disparities and living standards, landlessness, effects of environmental changes, technological revolution and social network structures and functions are commonly cited as the reasons for a household’s decision to migrate (Anarfi et al. 2003; Ziblim 2013c).

In Ghana, the three northern regions are considered the poorest, with high levels of food insecurity and malnutrition (Ghana Statistical Service 2012). Estimates show that about 80% of the population in the three northern regions is poor (Asante and Aikins 2008; Boateng and Awunyor-Vitor 2013; Adepoju 2008) and access to healthcare is known to be problematic. There is feminisation of poverty in the three northern regions (GSS 2012; Wrigley-Asante 2008; Awumbila and Ardayfio-Schandorf 2008; Doku et al. 1995). This implies that more women suffer from income poverty than men. As a result, both men and women from these poverty-prone communities migrate to the urban centres in the south to seek greener pastures and the associated economic gains. The Ghana Statistical Service (GSS) report (2012) shows that more than 56% of the migrant head porters are women and young school-age girls. Up to 84% of the *kayayoo* working in Kumasi (the major urban community and capital city of the Ashanti region) have migrated from the three northern regions while the others have migrated from neighbouring regions in Ghana and countries further afield (GSS 2012; PPVA 2011).

A major economic activity of the *kayayoo* is the carrying or transporting of goods for shoppers or traders in and around the commercial centres for a negotiated fee (Ziblim 2013c; Yeboah 2008). In Ghana, migrant female head porters have been tagged with different names within the social context that they operate their ‘*kaya’* business. In Kumasi, these migrants are called *‘paa-o-paa’,* and in Accra they are referred to as *‘kayayoo’* or *‘kayayei’. ‘Kayayoo*’ is the Ga terminology used to describe a woman who carries head loads for a negotiated fee. Etymologically, *‘kayayoo*’ comes from two languages, Hausa and Ga. In Hausa, *‘kaya’* means wares or goods, while *‘yoo’* in Ga means woman. Hence, *kayayoo* means a female head porter in Accra (Ziblim 2013c; Yeboah 2008; Yeboah and Appiah 2009).

The environment in which many of these head porters work is unconventional and unprotected from a variety of different hazards. Job and income insecurities are prominent features of the labour market (Apt and Amankrah 2004; Awumbila 2007). It must be emphasised that the majority of head porters (*kayayoo)* are unskilled and are relegated to a low status in the informal sector of the economy. They occupy the “three D’s” (dirty, dangerous and difficult) (ILO 2001). There is poor public and environmental health in both the residential and market areas in which the head porters operate, and this jeopardises the health of many of these informal workers (Ziblim 2013b; Apt and Amankrah 2004; World Bank 2008). Access to healthcare for these vulnerable migrants is limited in most cases.

However, it is a widely held belief that good health is associated with economic growth and development. For this reason, many governments of developing countries are combating poverty and boosting development through providing adequate access to healthcare (Yuansheng 2004). This is expressed by the declaration of the World Bank to the effect that access to healthcare is justified in economic terms by its benefit in improving the health of the entire community, leading to conditions that favour economic growth (World Bank 2009). In addition, the United Nations in its convention on economic, social and cultural rights recognises the right of everyone to enjoy the highest attainable standard of physical and mental health and requires governments to create conditions that assure all medical services and medical attention in the event of sickness (Richardson 1996).

In view of this, Ghana’s NHIS has become a much-needed attempt to address the challenge of extending social healthcare protection to all Ghanaians, especially the vulnerable groups. The conscious inclusion of informal workers such as female head porters into a nationwide health insurance scheme comes, perhaps, as no surprise considering that over 90% of Ghana’s workforce is in the informal economy (Heintz 2005). Again, the NHIS (designed as a replacement of a “cash-and-carry system”) is one of the social intervention schemes mitigating inequalities in healthcare (Gyasi 2015) because it seeks to make healthcare affordable and accessible. According to Mandersheid (2013), having health insurance is key to healthcare access. Therefore, the extent to which migrant female head porters are enrolled in and renew and utilise the NHIS determines their healthcare access in Ghana.

A number of factors affect the enrolment in and utilisation and renewal of the NHIS (Agyei-Baffour et al. 2013). These include health insurance coverage and cost, the nature of the provider, and physical or psychosocial distance (Amoah 2014). Parez et al. (2010) and Schoeps et al. (2011) accentuate this view by concluding that geographical access to healthcare facilities tends to influence health services usage. Similarly, Du et al. (2001) confirmed that long distances from health facilities lead to low demand for and use of healthcare services for the poor in general, and children in particular. They cause patients from rural areas to travel longer hours to visit health facilities than those in urban areas. Similarly, Buor (2008), in a study analysing socio-spatial inequalities in health services in sub-Saharan Africa confirms that distance plays a significant role in healthcare accessibility. According to Asah (2013), distance is a crucial determinant for enrolment in and utilisation and renewal of the NHIS by parents. This is related to the theory of distance decay, which states that things farther away are unlikely to be used (Basaza et al. 2008; Skov-Petersen 2001).

Several studies have also investigated the influence of socio-demographic factors on membership renewal and subscription to the NHIS (De Allegri et al. 2006a, Dalinjong and Laar 2012; Dong et al. 2009; Nketiah-Amponsah 2009, Sarpong et al. 2010). Again, Asah (2013) posits that income, age, education, long queues and the attitude of NHIS officials all affect enrolment, utilisation and renewal. Jehu-Appiah et al. (2011) buttressed this view by concluding that the NHIS price, benefits and convenience of administration are the most important factors and have the strongest association with enrolment and retention decisions. It has also been noted in other studies that a relationship exists between different demographic factors and NHIS enrolment and subsequent renewal of membership (De Allegri et al. 2006b; Sinha et al. 2006; Kamuzora and Gilson 2007; Ndiaye et al. 2007 and Asante and Aikins 2008).

The challenge of having access to healthcare can be limited by the amount and scope of coverage, as well as by the costs that one must bear. Although several studies have focused on the impact of the NHIS on the poor, no study appears to have specifically explored the enrolment in and utilisation and renewal of the NHIS by migrant female head porters in Kumasi Metropolis. This study, therefore, aimed to analyse the factors associated with female migrant head porters’ enrolment in and utilisation and renewal of NHIS in the central business district of Kumasi Metropolis.

## Materials and methods

### Head portering in Kumasi Metropolis

Head portering in Ghana is an ancient practice used to transport farm produce to market places for distribution (UNDP 2003). Head-load portering in market centres was introduced to Ghana from the Sahelian countries of Mali and Niger (Agyei et al. 2015). Head-portering activities play a significant economic role because many markets in Ghana are served by narrow, mud footpaths, which are not accessible by vehicle (Kwankye et al. 2009). It must be emphasised that women and children have played a prominent role in head portering since the 1969 Aliens Compliance Law, which resulted in the expulsion of illegal foreigners, many of whom had worked as *kayayoo* (Agyei et al. 2015). Although their ages range between 15 and 35 years (Baah-Ennumh et al. 2012), many of today’s *kayayoo* are young girls who have migrated from the north because of poverty, marriage pressures and lack of employment at home (Yeboah and Appiah 2009). The actual number of *kayayoo* in Ghana is unknown because there has been no national survey dedicated to providing data on them. However, Baah (2007) reported an estimated 23,000 *kayayoo* in Kumasi.

Female head porters face myriad difficulties such as violence, assault, verbal abuse, sexual harassment and exploitation by customers (Baah 2007). Many *kayayoo* work and sleep in market places, bus terminals, on streets or in front of stores and are exposed to the risk of sexual abuse, while others exchange sex for shelter (ILO 2005). This triggers the spread of sexually transmitted diseases such as HIV and increases the risk of teenage pregnancy, which has the potential to affect the porters’ health and that of their babies (Baah-Ennumh et al. 2012). In residences, female head porters share rooms at an average occupancy rate of four to five, living in wooden shacks or rented compound houses, which is above the maximum room occupancy of two persons per room as stipulated by the National Housing Policy (Ziblim 2013a). This also poses health threats (UN-HABITAT 2011).

Although a body of research shows that head porters experience diverse difficulties, there is an absence of social protection mechanisms such as access to healthcare, and this remains an important question for researchers.

### Theoretical framework

Various theories and models have explained the enrolment in and renewal and utilisation of the NHI Scheme, but this study is different in that it was conducted using the predisposing-enabling-need model (Andersen and Newman 1973). According to Andersen and Newman (1973), predisposing characteristics and socio-economic factors (comprising age, gender, religion, education and employment, marital status and number of children) influence an individual’s ability to utilise healthcare facilities in their prevalent situation. An individual is also more or less likely to use health services based on demographics, position within the social structure and belief in health services benefits. Enabling characteristics give one the ability to pay for healthcare services, either out-of-pocket or through health insurance. Need factors include the health status, nature of illness and perception (Andersen and Newman 1973). This study, however, only focused on the enabling income factor and the health status of the head porters.

Our study introduces several other important factors as well, namely the convenience and benefit factors in addition to the predisposing, enabling and need factors. Convenience factors relate to the ease with which a person can subscribe to the NHIS. These include distance to the NHIS office and time spent at the office before service delivery.

**Figure 1:**
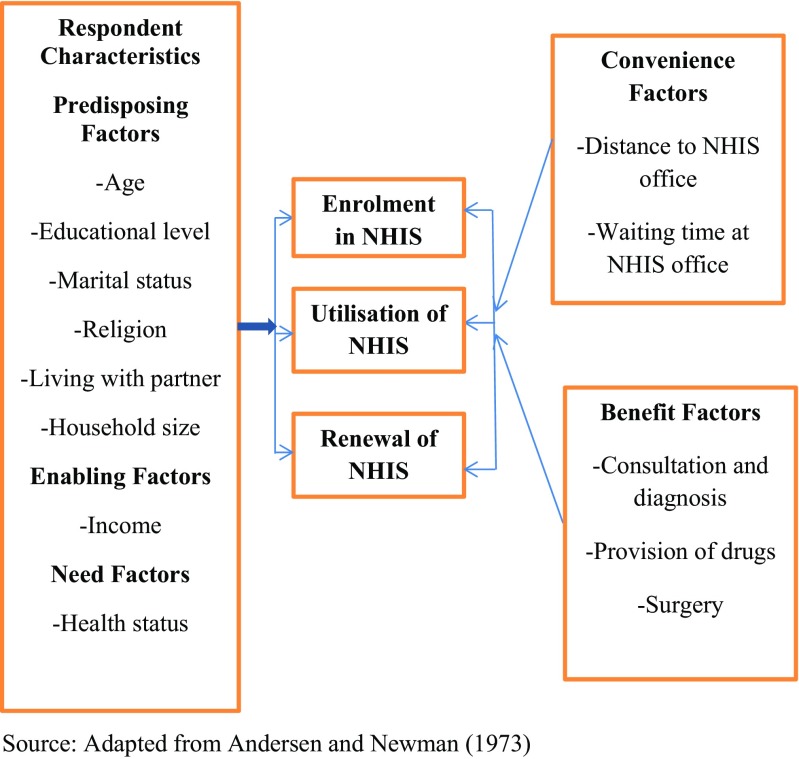
Conceptual framework. Source: Adapted from Andersen and Newman (1973)

Benefit factors are advantages accrued to an NHIS subscriber. These include consulting, dispensing of drugs and surgery (see Fig 1).

### Sampling design and data collection

We used both purposive and simple random sampling techniques to select the study areas and the migrant female head porters in those areas. Purposive sampling was used to select the study locations (Kejetia, Asafo and Bantema markets) (Freebody 2003). These locations were deliberately chosen because they are the largest market centres with intense head-portering activities. On the other hand, the simple random sampling technique was used to select respondents (female head porters). We used a structured questionnaire to collect the data. Since questionnaire administration took 2 weeks in three different market centres, the names of the sampled migrant female head porters were written down to ensure that every head porter had one chance to fill in the questionnaire. The structured questionnaire was used to gather standardised data for quantitative analysis (Bryman 2004) and we used the Kirkwood formula as in Eq. (1) to calculate the sample size (Kirkwood et al. 2003). The Kirkwood formula is explained in Eq. (1) as:

1 $$ n=\left[\frac{Z^2 pq}{e^2}\right] $$

Here: n = sample size; z = standard normal deviation 1.96; p = percentage of migrant female head porters in Kumasi Metropolis, estimated to be 56% (GSS 2012); q = 1.0-p and e = level of precision (95%). The minimum sample size was 378. However, an anticipated 10% non-response effect was added, arriving at the final sample size of 416. After sorting and verification of data, the study relied on 392 questionnaires for the analysis.

### Data analysis

Data were verified and entered into an electronic database and analysed statistically using SPSS software, version 21.0. Descriptive statistics were carried out to describe the background characteristics of the study sample. The chi-square (2) test was used to estimate the association of respondent characteristics, convenience factors and benefit factors with enrolment in and renewal and utilisation of the NHIS. Bivariate and multivariate analyses were used to further estimate the relationship between respondent characteristics and convenience and benefit factors with regard to enrolment, renewal and utilisation. We also used a binary logit regression model to estimate the associations among baseline characteristics, convenience and benefit factors, enrolment in and renewal and utilisation of the NHIS. The interpretation of the tests results considered an error margin of less than 5% as significant.

### Model specifications

Three more models were specified on the basis of Gujarati and Porter (2003):

2 $$ ENHIS={\propto}_0+{\propto}_1 AGE+{\propto}_2 MS+{\propto}_3 NYSCH+{\propto}_4 INC+{\propto}_5 HS+{\propto}_6R+{\propto}_7 HS+{\propto}_8 LWP+{\propto}_9 LNHIS+{\propto}_{10}\  TSNHIS+{\propto}_{11}\ C+{\propto}_{12} PD+{\propto}_{13}S+\varepsilon $$

3 $$ RENHIS={\propto}_0+{\propto}_1 AGE+{\propto}_2 MS+{\propto}_3 NYSCH+{\propto}_4 INC+{\propto}_5 HS+{\propto}_6R+{\propto}_7 HST+{\propto}_8 LWS+{\propto}_9 LNHIS+{\propto}_{10}\  TSNHIS+{\propto}_{11}\ C+{\propto}_{12} PD+{\propto}_{13}S+\varepsilon $$

4 $$ UNHIS={\propto}_0+{\propto}_1 AGE+{\propto}_2 MS+{\propto}_3 NYSCH+{\propto}_4 INC+{\propto}_5 HS+{\propto}_6R+{\propto}_7 HST+{\propto}_8 LWP+{\propto}_9 LNHIS+{\propto}_{10}\  TSNHIS+{\propto}_{11}\ C+{\propto}_{12} PD+{\propto}_{13}S+\varepsilon $$

Whereby:

ENHIS = probability that head porters would enrol in the NHIS; RNHIS = probability that head porters would renew their insurance as required by the scheme; UNHIS = probability that head porters would use the NHIS to access healthcare when sick. AGE, MS, NYSCH, INC, HS, R, HST, LWP, LNHIS, TSNHIS, C, PD and S correspond to age, marital status, number of years spent in school, income, household size, religion, health status, living with partner, location of NHIS office, time spent at NHIS office, consulting, provision of drugs and surgery respectively; α = parameters to be estimated; ε = error term.

The variables in Eqs. (2), (3) and (4) are described in Tables 1 and 2.

**Table 1:** Descriptions of respondent characteristics

Variables	Operational definition	Category	Code
Age	Age of respondent in years at time of study	-	-
Marital status	Marital status of head porter	Single	1
		Married	2
		Divorced	3
		Widowed	4
Level of education	Completed grade of schooling	Numerical	
Religion	Religious affiliation of respondent	Christianity	1
		Islam	2
Household size	Number of people in household	Numerical	
Living with partner	Living with partner at time of study	Yes	1
		No	0
Monthly household income	Monthly income earned from head portering and other economic activities	≤300	1
		301–500	2
		501–800	3
		801–1,000	4
Health status	Do you have any health problems?	Yes	2
		No	1

**Table 2:** Descriptions of variables

Convenience factors			
Location of offices	NHIS offices are conveniently located	Strongly disagree	1
		Disagree	2
		Agree	3
		Strongly agree	4
Time spent at NHIS office	NHIS times for card issuance are convenient	Strongly disagree	1
		Disagree	2
		Agree	3
		Strongly agree	4
Benefit factors			
Drugs provision	Required drugs are supplied for subscribers when accessing healthcare	Strongly disagree	1
		Disagree	2
		Agree	3
		Strongly agree	4
Consulting	Subscribers go through diagnosis before prescription	Strongly disagree	1
		Disagree	2
		Agree	3
		Strongly agree	4
Surgery	Accepted surgery under NHIS is provided free of charge when necessary	Strongly disagree	1
		Disagree	2
		Agree	3
		Strongly agree	4
Dependent variables			
Enrolment in NHIS	Is the head porter in the NHIS?	Yes	1
		No	0
Extent of enrolment in the NHIS	Annual renewal of NHIS card as required by scheme	Yes	1
		No	0
Utilisation of NHIS service	How often is healthcare sought with NHIS card?	Often	1
		Not often	0

## Results

### Bivariate analysis

The responses on enrolment in and renewal and utilisation of the NHIS are summarised in Table 3. The majority (78.8%) of the respondents had enrolled in the NHIS. Only 53.8% of these, however, were able to renew their NHIS cards annually as required by the scheme. The study found that 92.2% of the enrollees utilised the NHIS whenever they were afflicted by ill health.

**Table 3:** Responses on NHIS enrolment, renewal and utilisation

Responses	Initial enrolment in NHIS	Annual renewal of NHI	Utilisation of NHIS when sick
Yes	309 (78.83%)	180 (58.25%)	285 (92.23%)
No	83 (21.17%)	129 (41.75%)	24 (7.77%)

Source: field data

The responses on convenience and benefits factors are shown in Tables 4 and 5. The average distance from migrant female head porters’ residences to the nearest NHIS office was 2 km. The majority of head porters (59.8%) agreed with the statement that they spent an average of 8 h at NHIS offices when registering. The majority (75.2%) of participants were dissatisfied with provision of prescribed drugs, undergoing diagnosis (52.3%) and receiving surgery free of charge if required (65.0%) under the scheme.

**Table 4:** Chi-square test

Variable	Category	F (%) (N = 392)	Enrolment in NHIS (p-value) (N = 392)	Renewal of NHIS status (p-value) (N = 309)	Utilisation of NHI (p-value) (N = 309)
Age (years)	Minimum	18	0.018	0.001	0.004
	Maximum	44			
Marital status	Single	208 (53.1)	0.179	0.045	0.026
	Married	130 (33.2)			
	Divorced	27 (7.4)			
	Widowed	25 (6.3)			
No. of years of education	Minimum	0	0.077	0.012	0.038
	Maximum	12			
Religion	Christianity	261 (66.6)	0.488	0.572	0.775
	Islam	131 (33.4)			
Household size	<3	261 (66.6)	0.493	0.023	0.002
	3–5	53 (13.5)			
	>5	78 (19.9)			
Living with partner (N = 130)	Yes	19 (14.62)	0.519	0.410	0.692
	No	111 (85.38)			
Income	≤300	52 (13.3)	0.001	0.000	0.029
	301–500	182 (46.4)			
	501–800	132 (33.7)			
	801–1000	26 (6.6)			
Health status	Yes	54 (13.8)	0.231	0.023	0.001
	No	338 (86.2)			

Source: field data

**Table 5:** Chi-square test

Location of NHIS offices	Minimum	3 km	0.593	0.048	0.711
	Maximum	6.5 km			
Time spent at the NHIS office	Strongly disagree	114 (39.1)	0.349	0.031	0.122
	Disagree	81 (20.7)			
	Agree	152 (28.8)			
	Strongly agree	45 (11.4)			
Drug provision at the health facility	Strongly disagree	102 (26.0)	0.002	0.031	0.009
	Disagree	193 (49.2)			
	Agree	21 (5.4)			
	Strongly agree	76 (19.4)			
Consulting	Strongly disagree	93 (23.7)	0.017	0.011	0.025
	Disagree	112 (28.6)			
	Agree	129 (32.9)			
	Strongly agree	58 (14.8)			
Surgery	Strongly Disagree	97 (24.7)	0.233	0.004	0.000
	Disagree	158 (40.3)			
	Agree	104 (26.5)			
	Strongly agree	33 (8.5)			

*Pe, Pr and Pu = p-values for enrolment, renewal and utilisation respectively*

We estimated the association of respondent characteristics, convenience factors and benefit factors with NHIS enrolment, renewal and utilisation (see Table 5). Age (P_e_ = 0.018; P_r_ = 0.001; P_u_ =0.004) and income level (P_e_ = 0.001; P_r_ < 0.001; P_u_ = 0.029) significantly influenced NHIS enrolment, renewal and utilisation. Marital status (P_e_ = 0.179; P_r_ = 0.045; P_u_ = 0.026), number of years of education (P_e_ = 0.077; P_r_ = 0.012; P_u_ = 0.038) and household size (P_e_ = 0.493; P_r_ = 0.023; P_u_ = 0.002) highly significantly influenced renewal and utilisation of NHIS, but not enrolment. Religion and living with a partner also did not significantly influence enrolment in and renewal and utilisation of the NHIS.

Again, NHIS office location (P_e_ = 0.593; P_r_ = 0.048; P_u_ = 0.711) and time spent at the office (P_e_ = 0.349; P_r_ = 0.031; P_u_ = 0.122) significantly influenced renewal of the NHIS as required by the scheme but not the overall enrolment and utilisation of the NHIS. Consulting (P_e_ = 0.017; P_r_ = 0.011; P_u_ = 0.025) and provision of drugs (P_e_ = 0.002; P_r_ = 0.031; P_u_ = 0.009) significantly influenced enrolment in and renewal and utilisation of the scheme. Surgery (P_e_ = 0.233; P_r_ = 0.004; P_u_ = 0.000) significantly influenced renewal and utilisation of the NHIS but not enrolment.

The study produced the correlation coefficients between the dependent variables (enrolment in NHIS, renewal of NHIS card and utilisation of NHIS card) (Table 6). Enrolment in the NHIS had a weak but significant positive correlation with renewal of the NHIS card (p = 0.193) but a strong significant positive correlation with utilisation of the NHIS card (p = 0.684). Renewal of the NHIS card had a strong significant positive correlation with utilisation of the NHIS card (p = 0.984).

**Table 6:** Pearson’s correlation matrix

	Enrolment in NHIS	Renewal of NHIS card	Utilisation of NHIS card
Enrolment in NHIS	1.000	0.193**	0.684**
Renewal of NHIS card		1.000	0.984**
Utilisation of NHIS card			1.000

**Correlation significant at the 0.01 level (2-tailed)

### Multivariate analysis

In the multivariate analysis, we built three different models using binary logit regression (Table 7). Age and household income significantly increased migrant female head porters’ probability to enrol in, renew and utilise the NHIS. Married migrant head porters were significantly less likely to enrol in and renew the NHIS than single migrant head porters. However, married migrant head porters were also significantly more likely to utilise the NHIS when enrolled than single migrant female head porters. The number of years of education significantly increased the migrant female head porters’ likelihood to renew and utilise the NHIS. The household size of the migrant female head porters had a significant positive impact on enrolment in and utilisation of the NHIS, but a significant negative impact on the likelihood of renewing their NHIS status. Time spent at NHIS offices reduced the probability of enrolling in, renewing the membership of and utilising the NHIS, while drugs provided under the scheme significantly increased the odds of doing so. Consulting and surgery services significantly increased the likelihood to renew membership in and use the scheme, but not to enrol in it.

**Table 7:** Binary logit regressions results

Key variable	Variable	Enrolment in NHIS	Renewal of NHIS	Utilisation of NHIS
Respondent characteristics	Age (years)	0.553	0.354	0.581
		(21.400)*	(16.213)*	(21.860)*
	Married	-4.815	-2.111	4.858
		(8.217)*	(11.168)*	(8.029)*
	No. of years in school	-0.911	1.470	1.423
		(1.422)	(10.083)*	(5.591)*
	Household size	1.023	-0.408	1.033
		(4.731)*	(6.777)*	(4.514)*
	Income	0.020	0.011	0.021
		(9.909)*	(7.934)*	(9.400)*
	Health status	0.333	0.881	1.213
		(0.519)	(9.211)^*^	(10.312)^*^
Convenience factors	Location of NHIS office	0.179	0.020	0.154
		(0.284)	(0.005)	(0.205)
	Waiting time at NHIS office	-0.216	-0.168	-0.064
		(0.263)	(7.813)*	(1.022)
Benefit factors	Drug provision	0.106	0.027	0.224 (9.892)*
		(5.263)*	(0.523)*	
	Consulting	0.010	0.112	0.027
		(0.012)	(7.131)*	(8.195)*
	Surgery	0.125	0.095	0.181
		(0.269)	(6.001)*	(5.800)*
Number of observations		392	309	309
Cox and Snell R^2^		0.519	0.612	0.525
Nagelkerke R^2^		0.649	0.697	0.611
Omnibus test of model coeff. (χ^2^) Prob˃χ^2^		134.897 0.000	151.006 0.000	149.283 0.000

*Correlation significant at the 0.05 level

## Discussion

To the best of our knowledge, this is the first study investigating migrant female head porters’ enrolment in and utilisation and renewal of the NHIS in Ghana. The study found that the majority of migrant female head porters had enrolled in the NHIS. This may be attributed to a deliberate policy by the Ministry of Gender, Children and Social Protection (MGCSP) to enrol the vulnerable in the scheme for free, as a miniature of the Ghana Government’s commitment to mitigate the higher incidence of ‘cash and carry’ systems that previously existed in the healthcare delivery system. In addition, most enrollees utilised the scheme when afflicted with illness. However, there were some concerns about the NHIS since the scheme could not offer the quality healthcare needed. This corroborates the findings of Amoah 2014 and Asah 2013 in regard to the satisfaction of NHIS subscribers in Ghana.

Despite high initial enrolment, a substantial proportion of the head porters reported difficulties in renewing their NHIS status and were therefore denied access to healthcare. This provides evidence to suggest that head portering as an informal economic venture does not generate enough income to those involved to be able to renew their membership regularly, as required. Most people argue against the credibility of and the extent to which the NHIS provides good healthcare. This may result in fewer incentives for the head porters to engage in continual renewal of their insurance status, which is a matter of concern since sustainability of the scheme is partly dependent on the continuous renewal of the subscribers’ membership status. The disappointing renewal rate could also be explained by the long waiting times and delays that subscribers have to tolerate to reinstate their NHIS membership. This finding concurs with the observation of Gyasi (2015), which indicated that the NHIS does not offer value for money in terms of the health and well-being of its card bearers.

Age and economic status, using income level as a proxy, significantly influenced NHIS enrolment, renewal and utilisation. Older adults enrolled in and renewed and utilised the NHIS. This may be attributed to their vulnerability to various diseases. The study found that the household size of migrant female head porters has a negative significant impact on the probability to renew insurance, but a significant positive impact on the likelihood to utilise the scheme. Household size determines household consumption expenditure; hence, the larger the household is, the larger the household consumption expenditure. The study confirmed that household size has a negative impact on migrant female head porters’ probability to renew their insurance as required by the scheme. However, larger households also have more healthcare needs than smaller households, hence their propensity to use the scheme more when enrolled. These findings support previous studies such as those by Asah (2013), Dalinjong and Laar (2012), Dong et al. (2009), Nketiah-Amponsah (2009) and Sarpong et al. (2010) confirming that income and age affect NHIS enrolment, utilisation and renewal.

However, marital status, number of years of education and household size of the migrant female head porters significantly influenced renewal and utilisation of the NHIS, but not initial enrolment. Since the MGCSP registers the head porters free of charge, education levels did not have any significant influence on enrolment. Religion also did not significantly influence enrolment in or renewal and utilisation of the NHIS. Distance to NHIS offices and convenience of NHIS card issuance, however, did significantly influence NHIS renewal, but not enrolment and utilisation. The bureaucracy and stress the head porters go through in the process of enrolment demotivate them to enrol, as they cannot even afford to miss a day’s work. This could explain why the renewal rate in our sample was low and underpins empirical findings of other studies (Amoah 2014, Parez et al. 2010, Schoeps et al. 2011, Du et al. 2001, Buor 2008, Boateng and Awunyor-Vitor 2013, Sinha et al. 2006), which demonstrate that distance is a crucial determinant for NHIS enrolment, utilisation and renewal by subscribers.

The study also found that perceptions about the quality of drugs under the scheme significantly influenced enrolment, renewal and utilisation and revealed that consulting costs are usually not prohibitively expensive for the head porters, but drugs are. As a result, when the head porters realise that the NHIS cannot fully cover the cost of drugs dispensed at the hospital, they tend not to utilise it. Waiting times at the healthcare facility did not significantly influence NHIS enrolment, renewal and utilisation, as also confirmed by the report of Amoah (2014). Enrolment in the NHIS has a significantly weak positive correlation with renewal, but a strongly positive correlation with NHIS utilisation. NHIS renewal has a strong positive correlation with NHIS utilisation. This is because the main idea behind the subscription of head porters to the scheme is to enable them to access affordable healthcare in times of need. As long as it was associated with costs, however, head porters found it relatively difficult to renew their NHIS card.

## Conclusion and policy implications

This study revealed that implementation of the scheme has generally contributed to the female head porters’ NHIS enrolment, utilisation and, to some extent, renewal. Although the MGCSP registers the head porters free of charge, there are still a number of them who had not enrolled to benefit from the services of the scheme. Paradoxically, there were some respondents who had not enrolled, yet they could not afford to pay their hospital bills out-of-pocket either. Our study also found that socio-economic characteristics of migrant female head porters, such as income and age, significantly influence enrolment in the scheme.

This study reiterates that having insurance is not itself sufficient to access good healthcare because the insurance benefits may not adequately cover certain health services. The convenience and benefit factors contribute significantly to enrolment in and renewal of the NHIS. The study recommends an effective decentralisation of the National Health Insurance Scheme to the district level. Retail offices must be well equipped (computers, printers, cameras, etc.) to ensure that both enrolment and renewal processes can be done in a manner that reduces the inconvenience (long queues, long distances, wasting time, etc.) head porters go through during enrolment. Moreover, NHIS authorities should be using mobile vans and officers who will visit the subscribers, especially those vulnerable ones (female head porters) who live farther away from the NHIS offices to register them. This study again underscores the need to expand quality healthcare services (consulting services, providing of drugs, surgery) under the scheme so as to create a good public image and a positive perception about the scheme. NHIS authorities must examine the socio-economic characteristics of subscribers so as to create favourable environments that can promote enrolment in and renewal and utilisation of the scheme. In addition, policies enacted under the NHIS should take into consideration the vulnerable in society, such as female head porters, who are supposed to be the core beneficiaries.
